# Laminin 511-E8 Fragment Offers Superior Adhesion Properties for Gastric Cancer Cells Compared with Full-Length Laminin 511

**DOI:** 10.3390/cimb44040105

**Published:** 2022-04-05

**Authors:** Masaya Iwamuro, Hidenori Shiraha, Mayu Kobashi, Shigeru Horiguchi, Hiroyuki Okada

**Affiliations:** 1 Department of Gastroenterology and Hepatology, Okayama University Graduate School of Medicine, Dentistry and Pharmaceutical Sciences, Okayama 700-8558, Japan; hshiraha@gmail.com (H.S.); maaaaayu0917@gmail.com (M.K.); hiro@md.okayama-u.ac.jp (H.O.); 2 Department of General Medicine, Okayama University Graduate School of Medicine, Dentistry and Pharmaceutical Sciences, Okayama 700-8558, Japan; horiguchis@gmail.com

**Keywords:** cancer progression, extracellular matrix, gastric cancer cells, laminin 511-E8 fragment, laminin isoforms

## Abstract

**Simple Summary:**

Numerous studies over the past few decades have revealed that the interactions of gastric cancer cells with laminins through integrins play important roles in tumor cell proliferation, infiltration, and metastasis. However, the association between gastric cancer cells and the laminin E8 fragment, which is the smallest integrin-binding component, has not been investigated. In this study, we revealed that the laminin 511-E8 fragment had a greater impact on the adhesion, morphology, and proliferation of gastric cancer cells than full-length laminin 511. Thus, the laminin 511-E8 fragment is considered to be suitable for investigating the interaction between gastric cancer cells and extracellular matrices in tumor invasion and metastasis. Further, the involvement of Cdc42 in the laminin 511-E8 fragment-induced enhanced adhesion of gastric cancer cells was suggested.

**Abstract:**

Background: The interaction between cancer cells and laminin (Ln) is a key event in tumor invasion and metastasis. Previously, we determined the effect of full-length Ln511 on gastric cancer cells. However, the interactions between the Ln511-E8 fragment, a truncated protein of Ln511, and gastric cancer cells have not been investigated. Methods: We investigated the adhesion properties of gastric cancer cells to full-length Ln511 and Ln511-E8 fragments. Results: The proliferation of four gastric cancer cell lines (SH-10-TC, MKN74, SC-6-JCK, and MKN45) was highest on the Ln511-E8 fragment. Further, a larger cytoplasm was observed in SH-10-TC and MKN74 cells cultured on full-length Ln511 or Ln511-E8 fragments. The percentage of adhesive cells was highest on the Ln511-E8 fragment in all four cell lines. Moreover, adhesion of the gastric cancer cells to Ln511-E8 fragment-coated plates was reduced by the Cdc42 GTPase inhibitor in a dose-dependent manner, suggesting the involvement of Cdc42 in the Ln511-E8 fragment-induced enhanced adhesion of gastric cancer cells. Conclusions: The Ln511-E8 fragment had a greater impact on the adhesion, morphology, and proliferation of gastric cancer cells than full-length laminin. Thus, the Ln511-E8 fragment is suitable for investigating the interaction between gastric cancer cells and extracellular matrices in tumor invasion and metastasis.

## 1. Introduction

Laminins are large heterotrimeric proteins consisting of α, β, and γ subunits. Fifteen laminin isoforms were identified from combinations of five α-, four β-, and three γ-chains. For example, the α5β2γ1 heterotrimer isoform is defined as laminin 521. Although the biological functions of different chains and trimer molecules are largely unknown, numerous studies over the past few decades have revealed that the interactions of cells with laminins through integrins play important roles in tumor cell proliferation, infiltration, and metastasis [1,2,3,4]. In our previous study, we determined the effects of human recombinant laminin (Ln) 111, Ln121, Ln211, Ln221, Ln411, Ln421, Ln511, and Ln521 on gastric cancer cells [5], and revealed that the laminin isoform, Ln511, which consists of α5, β1, and γ1 chains, had a high affinity for gastric cancer cells, promoting cellular proliferation, increasing cell cytoplasmic volume, inhibiting invadopodia formation in some cells, and promoting the adhesion of cells to plates. Thus, we consider that Ln511 may be implicated in several stages of the tumor invasion and metastasis of gastric cancer cells.

In addition to full-length laminin isoforms, the laminin E8 fragment, which is the smallest integrin-binding component, has been used as a coating material for embryonic stem and induced pluripotent stem cell research for xeno-free, feeder-free, and sustained culture [6,7,8,9,10]. Laminin El’, E4, E8, and E3 fragments were first obtained via elastase digestion in full-length laminin [11,12]. Of them, the laminin E8 fragment, a 35-nm-long segment of the rod, is the terminal globule composed of the C-terminal regions of the α, β, and γ chains. It is known to be a functionally minimal form containing the active integrin-binding site but lacks other actions, such as heparin/heparan sulfate-binding activity [10,13,14,15]. Previous studies revealed that the Ln511-E8 fragment promotes greater adhesion in human embryonic and human-induced pluripotent stem cells compared with Matrigel and full-length Ln511 [10,13].

However, the effects of laminin E8 fragments on gastric cancer cells have never been investigated. In the present study, we focused on the Ln511-E8 fragment and sought to determine its effects on the adhesion, proliferation, and morphology of gastric cancer cells by comparing the properties of Ln511-E8 and full-length Ln511, based on the assumption that the truncated protein has a higher affinity for gastric cancer cells than full-length laminin.

## 2. Materials and Methods

### 2.1. Cell Lines and Reagents

The dishes or plates were coated with gelatin (Specialty media, Chemicon International, Burlington, MA, USA), human recombinant Ln511 (BioLamina Ab, Sundbyberg, Sweden), and Ln511-E8 fragment (iMatrix-511, Matrixome, Osaka, Japan) prior to cell seeding, according to the manufacturer’s instructions. The human gastric adenocarcinoma cell lines, MKN74 and MKN45, were purchased from the RIKEN BioResource Center (Ibaraki, Japan). SH-10-TC and SC-6-JCK cells were procured from the Cell Resource Center for Biomedical Research, Cell Bank (Miyagi, Japan), Institute of Development, Aging, and Cancer, Tohoku University. SH-10-TC cells were established from gastric mucinous adenocarcinoma, MKN74 cells from moderately differentiated tubular adenocarcinoma, and SC-6-JCK and MKN45 cells from poorly differentiated adenocarcinoma. Thus, MKN74 cells were classified as intestinal type, whereas SH-10-TC, SC-6-JCK, and MKN45 cells were classified as diffuse type according to the Lauren classification. Cells were grown in RPMI-1640 medium supplemented with 10% heat-inactivated fetal bovine serum (FBS), penicillin (5000 U/mL), and streptomycin (5 mg/mL) at 37 °C in a balanced air humidified incubator under a 5% CO_2_ atmosphere.

### 2.2. Western Blot Analysis

Gastric cancer cells were seeded in 60-mm dishes at a density of 3.0 × 10^5^ cells/dish. After incubation for 72 h, the cells were harvested and analyzed by Western blotting. Antibodies targeting E-cadherin (#14472; Cell Signaling Technology, Danvers, MA, USA), N-cadherin (#13116; Cell Signaling Technology), vimentin (#5741; Cell Signaling Technology), and β-actin (#4967; Cell Signaling Technology) were used as primary antibodies. HRP-conjugated anti-mouse IgG (#7076; Cell Signaling Technology) or anti-rabbit IgG antibodies (#7074; Cell Signaling Technology) were used as secondary antibodies.

### 2.3. Cellular Proliferation Assays

The proliferation of SH-10-TC, MKN74, and MKN45 cells was measured using a modified MTT [3-(4,5-dimethylthiazol-2-yl)-2,5-diphenyltetrazolium bromide] assay, which distinguishes live cells based on their ability to convert thiazolyl blue to dark blue formazan. Cells were seeded in 12-well culture plates at a density of approximately 5.0 × 10^5^ cells/plate. After incubation for 48 h at 37 °C in a balanced air humidified incubator under a 5% CO_2_ atmosphere, 50 µL of MTT was added to each well, and the reactions were incubated at 37 °C for 1 h. Subsequently, the medium with MTT was removed and 500 µL of DMSO was added to each well for 30 min at room temperature to solubilize the formazan product. Absorbance was measured at 570 nm using MULTISKAN GO (Thermo Fisher Scientific, Waltham, MA, USA).

The proliferation of SC-6-JCK cells was measured using an MTS [3-(4,5-dimethylthiazol-2-yl)-5-(3-carboxymethoxyphenyl)-2-(4-sulfophenyl)-2H-tetrazolium)] cell proliferation colorimetric assay kit (CellTiter 96^®^AQueous One Solution Cell Proliferation Assay, Promega, Madison, WI, USA). Cells were seeded in 12-well culture plates at a density of approximately 5.0 × 10^5^ cells/plate. After incubation for 48 h at 37 °C in a balanced air humidified incubator under a 5% CO_2_ atmosphere, 100 µL of MTS was added to each well, and the reactions were incubated at 37 °C for 1 h. Absorbance at 490 nm was measured using a MULTISKAN GO.

SH-10-TC, MKN74, SC-6-JCK, and MKN45 cell proliferation were measured using the Countess automated cell counter (Invitrogen, Waltham, MA, USA). The cells were seeded in six-well culture plates at a density of approximately 5.0 × 10^5^ cells/plate. After incubation for 48 h at 37 °C in a balanced air humidified incubator with 5% CO_2_, a single cell suspension was prepared using 0.25% trypsin-EDTA, and the number of cells was counted using the Countess automated cell counter.

### 2.4. Immunostaining

Gastric cancer cells were incubated for 48 h after seeding. Subsequently, the cells were washed twice with PBS and fixed in 4% paraformaldehyde for 20 min at 27 °C. After fixation, the cells were washed twice with PBS and then immersed in PBS containing 0.5% Triton X-100 (Nacalai Tesque, Kyoto, Japan) for 10 min at 27 °C. For triple staining with phalloidin, cortactin, and DAPI, cells were labeled with Acti-stain 488 phalloidin and rabbit anti-cortactin antibody (Abcam, #ab81208, Cambridge, UK), and stored overnight at 4 °C. After three washes with PBS containing 0.1% Triton X-100 at 3 min intervals, an Alexa Fluor 488-conjugated goat anti-rabbit IgG antibody (Molecular Probes, Thermo Fisher Scientific) was used as the secondary antibody. After three washes with PBS, the cells were stained with DAPI.

The cell area was measured using Photoshop Elements 13 software (Adobe, San Jose, CA, USA) by tracing the outline of the five representative cells.

### 2.5. Calculation of Adhesive Cells

Cell adhesion was measured using an MTS cell proliferation colorimetric assay kit (CellTiter 96^®^AQueous One Solution Cell Proliferation Assay). Cells were seeded in 96-well culture plates at a density of approximately 2500 cells/100 µL/well. Immediately after seeding and after incubation for 1, 3, 6, and 12 h at 37 °C in a balanced air humidified incubator under a 5% CO_2_ atmosphere, the medium was aspirated and washed with PBS to remove non-adherent cells. Subsequently, 100 µL of fresh medium and 20 µL of MTS were added to each well. Simultaneously, 20 µL of MTS was added to another three wells, without aspirating the medium, and used as a control. The reactions were incubated at 37 °C for 1 h, and absorbance at 490 nm was measured using a MULTISKAN GO. The percentage of adhesive cells was calculated by dividing the number of adhesive cells by the number of control cells.

### 2.6. Quantitative mRNA Expression Analysis

To explore the genes involved in gastric cancer cell adhesion to the Ln511-E8 fragment-coated plates, we used pre-designed 96-well microtiter plates. RNA from SH-10-TC cells cultured for 24 h on non-coated, Ln511-coated, and Ln511-E8 fragment-coated plates was extracted using QIAshredder (Qiagen, Venlo, The Netherlands) and RNeasy Mini Kits (Qiagen). Complementary DNA was prepared from 2 μg of total RNA using MuLV reverse transcriptase (Applied Biosystems, Waltham, MA, USA) and RNase inhibitor (Applied Biosystems). TaqMan array plates (No. 4418796, human ILK signaling, Thermo Fisher Scientific) and StepOnePlus real-time PCR system (Thermo Fisher Scientific) were used according to the manufacturer’s instructions. Quantitative measurements were performed, and a heatmap was generated using the ExpressionSuite software (Thermo Fisher Scientific).

### 2.7. Blocking Study

To investigate the possible involvement of Cdc42 in superior cell adhesion to the Ln511-E8 fragment, we tested the adhesion-blocking ability of the Cdc42/Rac1 GTPase inhibitor (Sigma Aldrich, Burlington, MA, USA), a selective, reversible, non-competitive inhibitor of Cdc42 GTPases. We added 0, 1, 10 or 100 µM of Cdc42 GTPase inhibitor when cells were seeded in 12-well culture plates at a density of approximately 5.0 × 10^5^ cells/plate. After incubation for 1 h at 37 °C in a balanced air humidified incubator under a 5% CO_2_ atmosphere, the medium was aspirated and washed with PBS. The number of SH-10-TC, MKN74, and MKN45 cells adhered to the plates was measured using an MTT assay, and that of SC-6-JCK cells was measured using an MTS assay, as described above.

We also tested the adhesion-blocking ability of the Rac1 inhibitor (Sigma Aldrich), which is a reversible inhibitor of Rac1 GDP/GTP exchange that interferes with the interaction between Rac1 and Rac-specific guanine nucleotide exchange factors Trio and Tiam1, and the Rho inhibitor Rhosin (Merck, Darmstadt, Germany), which is known to bind to Trp58 of RhoA, inhibit its activation by Rho guanine nucleotide exchange factors, and inhibit RhoC activation. The cells were seeded in 12-well culture plates at a density of approximately 5.0 × 10^5^ cells/plate, and 0, 10, 50 or 250 µM Rac1 inhibitor or 0, 3, 30 or 300 µM Rho inhibitor was added. After incubation for 1 h, cell adhesion was evaluated as described above.

### 2.8. Statistical Analysis

Numerical values are expressed as mean ± standard deviation. Data are representative of three independent experiments, unless the number of samples is indicated. Student’s *t*-test was used to compare the two population means. For multiple comparisons, statistical analysis was performed using one-way analysis of variance, followed by Tukey–Kramer post hoc test. Statistical analyses were performed using JMP 14.0.0 software (SAS Institute, Cary, NC, USA).

## 3. Results

### 3.1. Mesenchymal/Epithelial Phenotypes of the Gastric Cancer Cell Lines Tested

**Figure 1:**
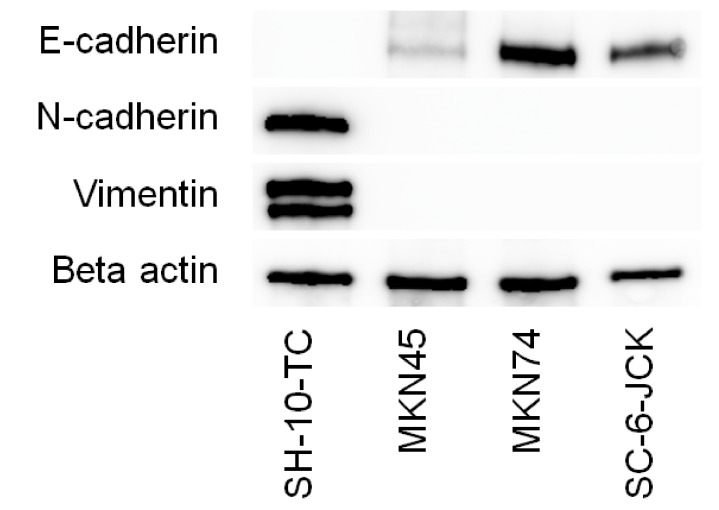
Phenotyping of SH-10-TC, MKN45, MKN74, and SC-6-JCK cells by immunoblot analysis. Cells were cultured for 72 h, lysed, and evaluated via Western blot analysis. SH-10-TC cells possessed a mesenchymal phenotype, with negative expression for E-cadherin and positive expression for N-cadherin and vimentin. Conversely, MKN45, MKN74, and SC-6-JCK cells possessed an epithelial phenotype, with positive expression for E-cadherin and negative expression for N-cadherin and vimentin.

Western blot analysis of four gastric cancer cell lines (Figure 1) revealed that SH-10-TC cells possessed a mesenchymal phenotype, as they were positive for N-cadherin and vimentin, and negative for E-cadherin. Conversely, MKN45, MKN74, and SC-6-JCK cells possessed an epithelial phenotype, with positive expression of E-cadherin and negative expression of N-cadherin and vimentin. These results align with those of our previous study [5].

### 3.2. Ln511-E8 Fragment Promotes Gastric Cancer Cell Proliferation

**Figure 2:**
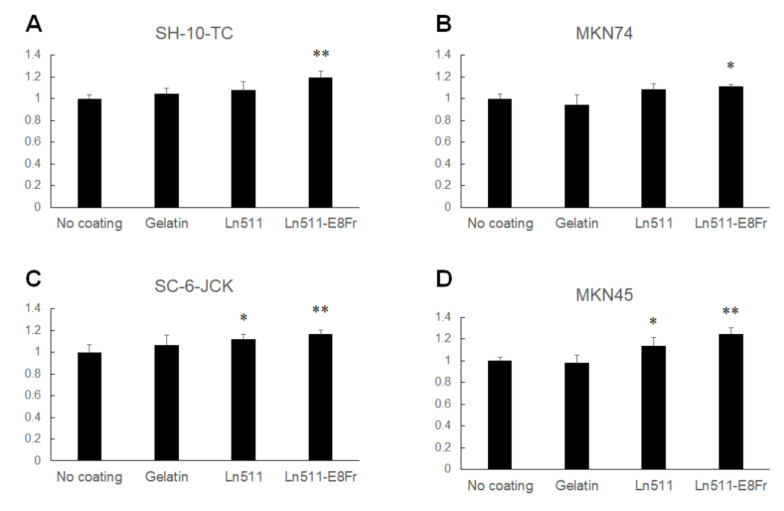
Results of the MTT/MTS assay. The proliferation of SH-10-TC gastric cancer cells cultured for 48 h is significantly higher on the Ln511-E8 fragment than on the control (no coating) (**A**). MKN74 cells display greater proliferation on the Ln511-E8 fragment than on the control (no coating) (**B**). Although markedly larger proliferative responses of SC-6-JCK (**C**) and MKN 45 cells (**D**) were found on the full-length Ln511 and Ln511-E8 fragments than on the control, the number of cells was largest when cultured for 48 h on the Ln511-E8 fragment. These results indicate that Ln511-E8 fragment promotes gastric cancer cell proliferation. * *p* < 0.05, ** *p* < 0.01, as indicated (vs. no coating). Data are presented as the mean ± standard deviation of three independent experiments.

Figure 2 shows the relative cell numbers of gastric cancer cells cultured for 48 h without coating (control) or on gelatin-, Ln511-, or Ln511 E8 fragment-coated plates. The cell numbers of SH-10-TC (119.3% ± 6.0%), MKN74 (111.6% ± 1.5%), SC-6-JCK (116.6% ± 4.1%) and MKN45 (124.7% ± 5.6%) were the largest in Ln511 E8 fragment-coated wells, and the differences were significant compared to the cells in the controls. Although the number was lower than that of cells in the Ln511 E8 fragment-coated wells, SC-6-JCK (112.0% ± 4.4%) and MKN45 cells (113.8% ± 7.6%), cultured in Ln511-coated wells, were significantly increased compared to cells in the controls.

**Figure 3:**
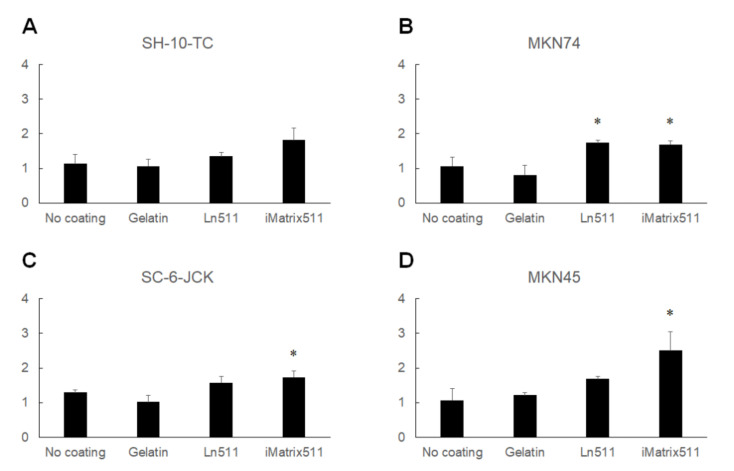
Results of cell counting. Although the difference was not significant, the number of SH-10-TC gastric cancer cells cultured for 48 h was the highest on the Ln511-E8 fragment (**A**). MKN74 (**B**), SC-6-JCK (**C**), and MKN45 cells (**D**) showed significantly greater proliferation on the Ln511-E8 fragment than on the control (no coating). * *p* < 0.05, as indicated (vs. no coating). Data are presented as the mean ± standard deviation of three independent experiments.

The results of direct cell counting using an automated cell counter are shown in Figure 3. Although the difference was not significant, the cell count of SH-10-TC cultured on Ln511-E8 fragment-coated plates was the highest (182.2% ± 33.6%). MKN74 (169.4% ± 9.3%), SC-6-JCK (173.5% ± 17.8%) and MKN45 cells (250.0% ± 55.1%) showed significantly greater proliferation on the Ln511-E8 fragment than on the control (no coating). These results indicated that the Ln511-E8 fragment promoted the proliferation of all four gastric cancer cell lines.

### 3.3. Ln511 and Ln511-E8 Fragments Modulate the Morphology of Some Gastric Cancer Cells

**Figure 4:**
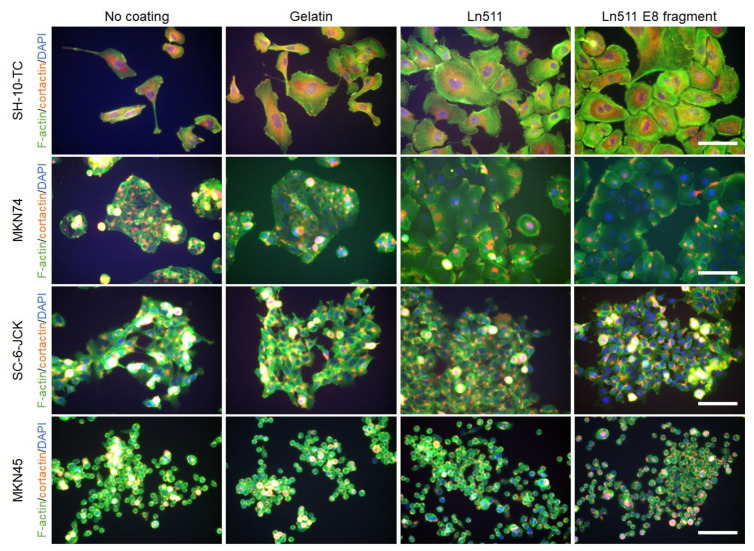
Morphologies of gastric cancer cells cultured for 48 h. Phalloidin-actin (green), cortactin (red), and DAPI (blue) staining showed that the cytoplasm of SH-10-TC cells is the largest when cultured on full-length Ln511 or Ln511-E8 fragment (the top row). Larger cytoplasm was observed in MN74 cells cultured on full-length Ln511 or Ln511-E8 fragment (the second row). Morphology of SC-6-JCK (the third row) and MKN45 cells (the bottom row) did not differ between control (no coating) and cells cultured on full-length Ln511 or Ln511-E8 fragment. Scale bars = 100 μm.

**Figure 5:**
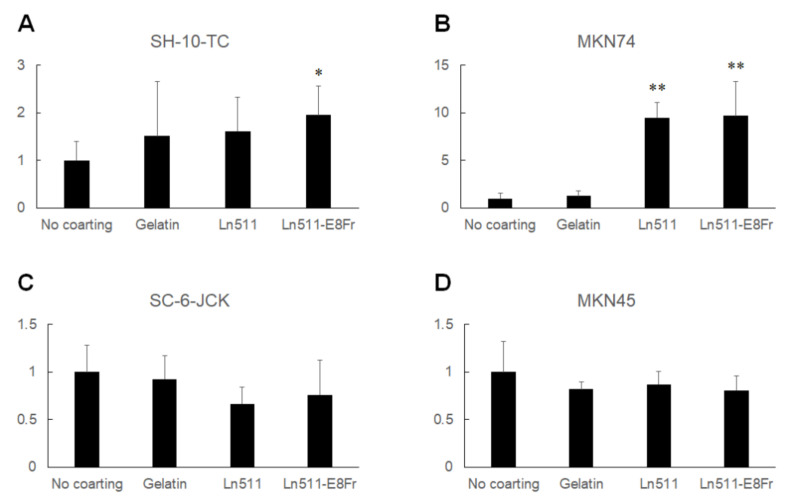
Difference in gastric cancer cell areas between the coating materials. Although the cytoplasm of SH-10-TC was larger on gelatin-, Ln511-, and Ln511-E8 fragment-coated plates than the control (no coating), the difference was not statistically significant for cells cultured on gelatin and Ln511, which might be due to the large standard deviation (**A**). The cell areas of MKN74 were apparently larger on the Ln511 and Ln511-E8 fragments than those of the control (no coating) (**B**). No differences in the sizes of the SC-6-JCK (**C**) and MKN45 cells (**D**) were observed. * *p* < 0.05, ** *p* < 0.01, as indicated (vs. no coating). Values are normalized to those of controls (no coating) and data are presented as mean ± standard deviation of five representative cells.

Triple staining results for phalloidin (green), cortactin (red) and DAPI (blue) are shown in Figure 4. As indicated in the top row, the cytoplasm of SH-10-TC cells was larger when cells were cultured on gelatin (151.3% ± 113.8%), Ln511 (161.2% ± 71.9%), or the Ln511-E8 fragment (195.5% ± 60.8%), compared with the control. The difference between the cell areas in the controls and those of the Ln511-E8 fragment was significant (Figure 5A).

Morphological changes were most prominent in MKN74 cells, as shown in the second row of Figure 4. The cytoplasm of the cells cultured on the Ln511 (947.8% ± 159.1%) and Ln511-E8 fragment (967.3% ± 363.4%) was significantly larger than that of the controls (Figure 5B).

No major changes were found in the cell areas in SC-6-JCK (Figure 5C) and MKN45 cells (Figure 5D). These results indicate that Ln511 and Ln511-E8 fragments alter the morphology of some gastric cancer cells. In particular, the Ln511-E8 fragment showed more prominent alteration of SH-10-TC cells than full-length Ln511.

### 3.4. Ln511-E8 Fragment Augments the Adhesion of Gastric Cancer Cells

**Figure 6:**
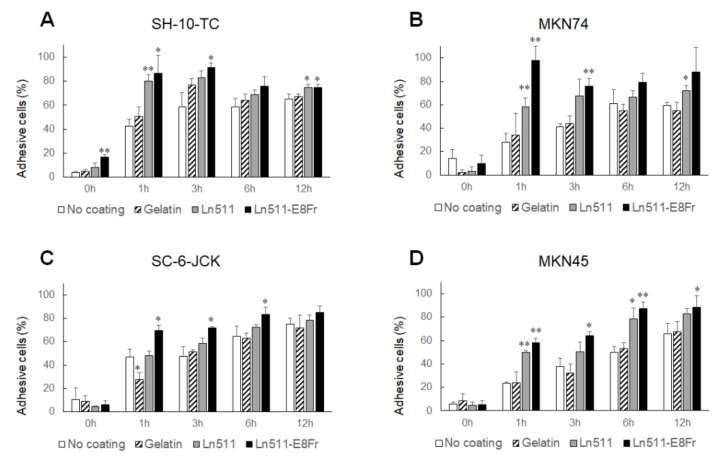
Results of cellular adhesiveness. Adhered cells were determined by the MTT/MTS assay. The percentages of adhesive SH-10-TC (**A**), MKN74 (**B**), SC-6-JCK (**C**), and MKN45 cells (**D**) were highest on the Ln511-E8 fragment. These results indicate the superior affinity interactions between gastric cancer cells and the Ln511-E8 fragment, rather than full-length Ln511. * *p* < 0.05, ** *p* < 0.01, as indicated (vs. no coating). Data are presented as the mean ± standard deviation of three independent experiments.

The percentage of adhered SH-10-TC cells is shown in Figure 6A. Without coating, 42.4 ± 5.9% of SH-10-TC cells adhered to the plate at 1 h, and 58.5 ± 12.0% of cells adhered to the plate at 3 h. At 12 h, 58.5 ± 7.4% of cells ultimately adhered to the plate. Gelatin coating did not affect the adhesion of SH-10-TC cells. In contrast, SH-10-TC cells cultured on Ln511 exhibited superior adhesiveness at 1 h (80.2 ± 5.2%) and 12 h (74.7 ± 2.9%) compared to the control (no coating). Similarly, the percentage of adhesive cells was higher on the Ln511-E8 fragment at 1 h (86.7 ± 14.9%), 3 h (91.5 ± 3.6%) and 12 h (74.6 ± 2.9%). Notably, 16.6 ± 2.1% of SH-10-TC cells were attached even at 0 h, suggesting prompt adhesion on Ln511-E8 fragment-coated plates.

The percentages of adhered MKN74 cells to non-coated plates at 1, 3 and 12 h were 27.9 ± 7.7%, 40.9 ± 2.9% and 59.5 ± 2.8%, respectively (Figure 6B). Coating the plates with Ln511 increased the adhesiveness of MKN74 to 58.2 ± 7.9% at 1 h and 71.9 ± 4.8% at 12 h. The adhesiveness of these cells to the Ln511-E8 fragment-coated plates was excellent, showing 97.7 ± 12.1% at 1 h and 75.8 ± 6.7% at 3 h. Similarly, MKN45 cells displayed the highest adhesive ability on Ln511-E8 fragment-coated plates, compared with non-coated, or gelatin- or Ln511-coated plates (Figure 6D).

Although Ln511 had no positive effect on the adhesiveness of SC-6-JCK cells to plates (Figure 6C), coating with the Ln511-E8 fragment showed superior adhesiveness at 1 h (69.5 ± 4.2%), 3 h (71.9 ± 0.85%) and 6 h (83.2 ± 6.1%), compared with the control (46.9 ± 6.6% at 1 h, 47.6 ± 8.1% at 3 h and 64.6% ± 8.9% at 6 h). Taken together, these results indicate that gastric cancer cells possess the highest affinity for the Ln511-E8 fragment.

### 3.5. Possible Involvement of Cdc42 in the Ln511-E8 Fragment-Induced Enhanced Adhesion of Gastric Cancer Cells

**Figure 7:**
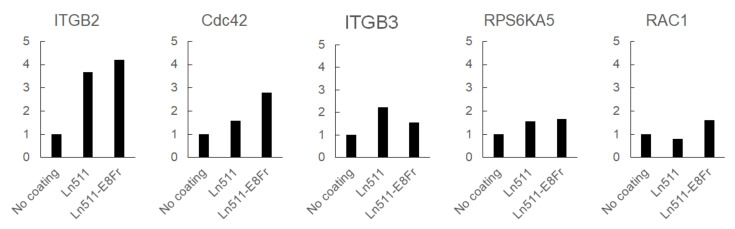
The five most upregulated genes in SH-10-TC cells cultured on Ln511 or Ln511-E8 fragment compared with the control (no coating) based on 96-well plate-based gene expression analysis. The difference in mRNA expression between control (no coating) and other samples was largest for integrin subunit beta 2 (ITGB2), followed by Cdc42, integrin subunit beta 3 (ITGB3), ribosomal protein S6 kinase A5 (RPS6KA5), and RAS-related C3 botulinus toxin substrate 1 (RAC1). Data were obtained from a single experiment using gene expression array plates.

We explored the factors involved in the superior adhesion of gastric cancer cells to the Ln511-E8 fragment-coated plates. The results of the 96-well plate-based gene expression analysis are shown in Supplementary Figure S1. The five most upregulated genes in SH-10-TC cells cultured on the Ln511 or Ln511-E8 fragment compared with no coating were integrin subunit beta 2 (ITGB2), followed by Cdc42, integrin subunit beta 3 (ITGB3), ribosomal protein S6 kinase A5 (RPS6KA5), and RAS-related C3 botulinus toxin substrate 1 (RAC1) (Figure 7). Based on the gene expression results, we focused on Cdc42, which is known to promote filopodia formation. We also investigated the involvement of Rac1, which promotes lamellipodia formation, and Rho, which is implicated in the formation of actin stress fibers and focal adhesions.

**Figure 8:**
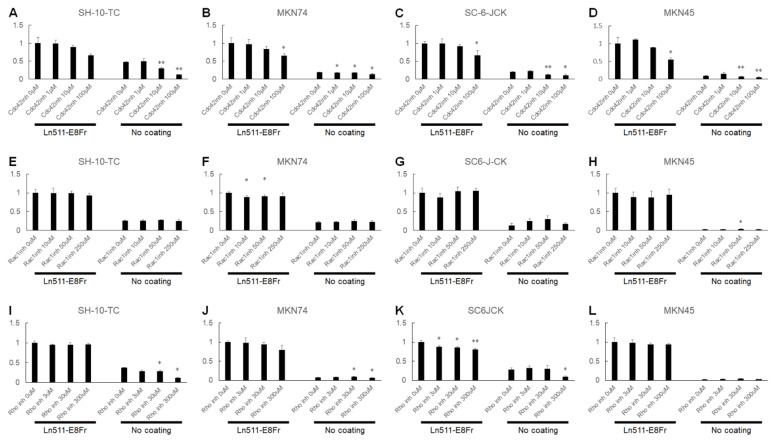
The percentages of adhesive cells at 1 h after seeding in the presence of the Cdc42 GTPase, Rac1, or Rho inhibitors. Adhesion of SH-10-TC (**A**), MKN74 (**B**), SC-6-JCK (**C**), and MKN45 cells (**D**) to the Ln511-E8 fragment-coated plates was reduced owing to the Cdc42 GTPase inhibitor in a dose-dependent manner, suggesting possible involvement of Cdc42 in superior gastric cancer adhesion to the Ln511-E8 fragment. The Rac1 inhibitor had no inhibitory effects on cell adhesion (**E**–**H**). The Rho inhibitor resulted in dose-dependent decrease in adhesion of SC-6-JCK cells (**K**). The Rho inhibitor had no inhibitory effects on cell adhesion of SH-10-TC (**I**), MKN74 (**J**), or MKN45 cells (**L**). * *p* < 0.05, ** *p* < 0.01, as indicated (vs. no coating). Data are presented as the mean ± standard deviation of three independent experiments. Values are normalized to those of controls (no coating).

As shown in Figure 8A, although the difference was not significant, the adhesion of SH-10-TC cells to the Ln511-E8 fragment coated wells was reduced in the presence of the Cdc42 GTPase inhibitor in a dose-dependent manner, showing an adhesion efficiency of 66.2 ± 4.4% with 100 µM of Cdc42 GTPase inhibitor, compared to cells without the inhibitor. The adhesion of MKN74 (64.9 ± 6.6%), SC-6-JCK (66.8 ± 12.9%) and MKN45 cells (54.4 ± 5.1%) was significantly hampered with 100 µM of Cdc42 GTPase inhibitor (Figure 8B–D). In contrast, the Rac1 inhibitor had no inhibitory effect on cell adhesion (Figure 8E–H). The Rho inhibitor resulted in a dose-dependent decrease in the adhesion of SC-6-JCK cells (80.7 ± 3.5% with 300 µM Rho inhibitor; Figure 8K). MKN74 showed a dose-dependent decrease in adhesion to the Ln511-E8 fragment-coated wells in the presence of the Rho inhibitor (Figure 8J), while the other two cell lines were not affected (Figure 8I,L). As Cdc42 GTPase inhibitors are a selective, reversible, non-competitive inhibitor of Cdc42 GTPases, these results indicate the possible involvement of Cdc42 in the Ln511-E8 fragment-induced enhanced adhesion of gastric cancer cells, whereas Rac1 and Rho are not or only partially involved.

## 4. Discussion

In this study, we characterized the superior adhesion of gastric cancer to the Ln511-E8 fragment, which is generally used in stem cell culture. Our results revealed that:

  i. The Ln511-E8 fragment promotes the proliferation of SH-10-TC, MKN74, SC-6-JCK, and MKN45 cells;
 ii. The cytoplasmic areas of gastric cancer cells cultured on the Ln511-E8 fragment were significantly larger than those without the coating material in SH-10-TC and MKN74 cells;
iii. SH-10-TC, MKN74, SC-6-JCK, and MKN45 cells promptly adhered to Ln511-E8 fragment-coated plates.

Rho family small G proteins play a central role in the regulation of the intracellular actin cytoskeleton and microtubule dynamics. Among the Rho family proteins in mammals, Cdc42, Rac1, and RhoA are the most extensively studied. Cdc42 promotes actin polymerization by the Arp2/3 complex by binding to and activating the effector protein, N-WASP [16,17]. Cdc42 can also activate mDia2 to induce actin polymerization independently of the Arp2/3-N-WASP pathway and regulate the severing of actin filaments through the PAK1-LIMK-cofilin pathway [18]. Cdc42 promotes bundling of actin filaments via the target proteins, mDia2 and N-WASP, leading to the formation of filopodia. Rac1 also forms a network of branched actin fibers via the WAVE regulatory complex and produces lamellipodia. RhoA forms actin fibrils via the target protein, mDia1, and induces actin–myosin interactions via Rho-kinase ROCK, resulting in the formation of stress fibers and focal adhesions. Our experiments using Cdc42-, Rac1-, and Rho-blocking agents revealed that the Cdc42 GTPase inhibitor resulted in a dose-dependent decrease in cell attachment of all four gastric cancer cell lines. In contrast, the Rho inhibitor did lead to differences in cellular attachment of SH-10-TC or MKN45 cells, and the Rac1 inhibitor had no effect on any of the cells. The effect of inhibition might not be specific for Cdc42 GTPase activity, since the concentrations of treated Cdc42 GTPase inhibitors were quite high. However, based on our results, Cdc42 may be involved in the Ln511-E8 fragment-induced enhanced adhesion of gastric cancer cells.

Filopodia tips serve as initial adhesion sites and act as nucleation cores to form lamellipodia for cells to spread efficiently [19]. As shown in the present study, SH-10-TC and MKN74 gastric cancer cells changed their morphology to cells with wider cytoplasm on the Ln511-E8 fragment-coated plates (Figure 4 and Figure 5). Such alterations in morphology, in addition to the prompt adhesion of gastric cancer cells (Figure 6), may be due to complete circumferential cell adhesion to the plate, which was augmented through Cdc42 in the presence of the Ln511-E8 fragment.

Chang et al. investigated RhoA activity in patients with gastric cancer and showed that high RhoA activity was associated with significantly worse overall survival in patients with diffuse-type gastric cancer [20]. In contrast, in patients with intestinal-type gastric cancer, no difference was observed in the overall survival between those with high and low RhoA activity. In the present study, the Rho inhibitor affected cell attachment in one of the diffuse-type cancers, such as SC-6-JCK (poorly differentiated adenocarcinoma), while the other two diffuse-type cancers, MKN45 (poorly differentiated adenocarcinoma) and SH-10-TC (mucinous adenocarcinoma), were not affected. Such discrepancies may be due to differences between the in vitro and in vivo studies. In this context, the role of Cdc42 in tumor invasion and metastasis of gastric cancer cells should be investigated in specimens derived from patients with cancer.

To date, 15 laminin isoforms have been identified in vivo. Each isoform has a different influence on cell differentiation, migration, and adhesion. For instance, during the differentiation of human-induced pluripotent stem cells into ocular lineages, the Ln211-E8 fragment leads to the formation of neural crest cells, and the Ln332-E8 fragment produces epithelial cells, including the corneal epithelium. The Ln511-E8 fragment induces ocular cell lineages, including corneal, retinal, and neural crest cells, mimicking in vivo eye development [21]. Our previous study revealed that in gastric cancer cells, Ln411 and Ln511 dynamically modulate proliferation, adhesion, and morphology, in a distinct manner; Ln511 displayed high affinity for gastric cancer cells and promoted static behavior, while Ln411 provided dynamic properties to some cell lines [5]. The present study further revealed that the Ln511-E8 fragment strongly promotes the adhesion and proliferation of gastric cancer cells, even compared with full-length Ln511.

## 5. Conclusions

We revealed that the Ln511-E8 fragment had a greater impact on the adhesion, morphology, and proliferation of gastric cancer cells than full-length laminin. Thus, the Ln511-E8 fragment is considered to be suitable for investigating the interaction between gastric cancer cells and extracellular matrices in tumor invasion and metastasis.

## Data Availability

The data supporting the findings of this study are available from the corresponding author, M.I., upon reasonable request.

## Supplementary Materials

The following supporting information can be downloaded at https://www.mdpi.com/article/10.3390/cimb44040105/s1, Figure S1: Heatmap showing the mRNA expression in SH-10-TC cells cultured for 24 h on non-coated, Ln511-coated, and Ln511-E8 fragment-coated plates.
