# Supplementary figures and images for: Laminin 511-E8 Fragment Offers Superior Adhesion Properties for Gastric Cancer Cells Compared with Full-Length Laminin 511
Source: Curr Issues Mol Biol. 2022 Apr 5;44(4):1539–51. doi: 10.3390/cimb44040105 (PMC9164087; doi:10.3390/cimb44040105)

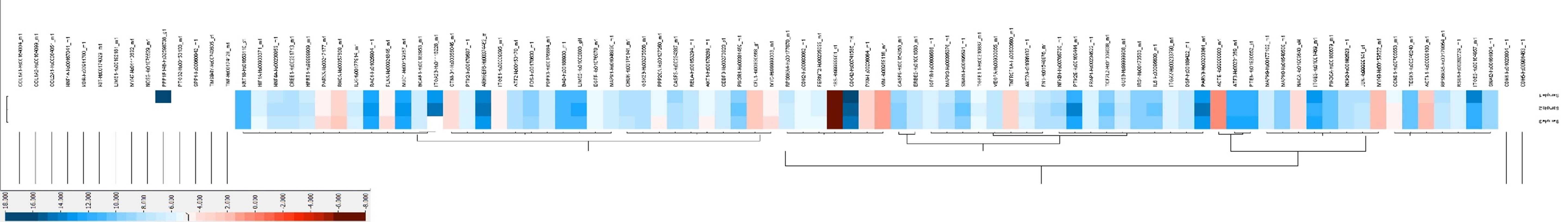

Supplement: Supplementary file 1 [file cimb-44-00105-s001.zip › cimb-1634264-supplementary/Supplemetary Figure S1.tif]
